# Manipulation under anesthesia after primary knee arthroplasty in Sweden: incidence, patient characteristics and risk of revision

**DOI:** 10.1080/17453674.2019.1637177

**Published:** 2019-07-04

**Authors:** Hunbogi Thorsteinsson, Margareta Hedström, Otto Robertsson, Natalie Lundin, Annette W-Dahl

**Affiliations:** a Department of Orthopedics, Skane University Hospital, Lund;; b The Swedish Knee Arthroplasty Register;; c Department of Orthopedics, Karolinska University Hospital, Huddinge;; d Department of Clinical Science, Intervention and Technology, Division of Orthopaedics and Biotechnology, Karolinska Institutet, Stockholm, Sweden

## Abstract

Background and purpose — The incidence of manipulation under anesthesia (MUA) after knee arthroplasty surgery has been reported to vary between 0.5% and 10%. We evaluated the incidence of MUA after primary knee arthroplasty in Sweden, the demographics of the patients and the risk of revision.

Patients and methods — Between 2009 and 2013, 64,840 primary total and unicompartmental knee arthroplasties (TKA and UKA) were registered in the Swedish Knee Arthroplasty Register (SKAR). MUAs performed between 2009 and 2014 were identified through the in- and outpatient registers of the Swedish National Board of Health and Welfare. Pertinent data were verified through medical records and patient demographics and revisions were obtained from the SKAR.

Results — 1,258 MUAs were identified. Of these, 1,078 were 1st-time MUAs, performed within 1 year after the primary knee arthroplasty. The incidence of MUA was 1.7% and the incidence varied between hospitals from 0% to 5%. The majority were performed after TKA (98%), in younger patients (65% < 65 years), women (64%), and relatively healthy persons (88% had ASA ≤ 2). The cumulative risk of revision at 10 years was 10% (95% CI 8.6–12), similar for men and women.

Interpretation — In Sweden, MUA is a rather uncommon measure after knee arthroplasty, especially after UKA. The CRR at 10 years was doubled compared to the general knee arthroplasty population. The frequency of the procedure varies between hospitals but in general it is performed more frequently in healthier and younger patients.

Joint stiffness following knee arthroplasty is a disabling complication. One treatment option is manipulation under anesthesia (MUA). However, the literature describes no clear definition/consensus of stiffness or the indications for MUA. Numerous potential risk factors have been reported for insufficient knee range of motion (ROM) after knee arthroplasty, among others younger age (Issa et al. 2015, Werner et al. 2015, Plate et al. 2016, Newman et al. 2018), female sex (Gadinsky et al. 2011, Werner et al. 2015), ethnicity (Issa et al. 2015), high BMI (Gadinsky et al. 2011), smoking (Werner et al. 2015, Issa et al. 2015, Newman et al. 2018), comorbidities such as diabetes (Plate et al. 2016), warfarin treatment (Desai et al. 2014), history of previous knee surgery (Plate et al. 2016, Newman et al. 2018), and limited preoperative ROM (Ritter et al. 2003, Kim et al. 2004, Keating et al. 2007, Issa et al. 2015, Newman et al. 2018).

The incidence of MUA after knee arthroplasty surgery has been reported to range from 0.5% to almost 10% (Table 1, see Supplementary data). Most were single-center studies performed in the United States with relatively few patients. 2 large US studies (Pfefferle et al. 2014, Werner et al. 2015) were based on the PearlDiver database (publicly available including private payers and Medicare data) and the Explorys database (commercially available including electronic healthcare data) but these showed different MUA incidence (1.5% and 4.3% respectively). A Finnish study reported on the incidence of MUA in 1 hospital during the first 6 months after the primary knee arthroplasty surgery, before and after implementing fast-track (Pamilo et al. 2018). They found a similar incidence before (2009–2010) and after (2012–2013) fast-track (6%). A Danish study on 359 TKAs was also performed in a fast-track hospital and found the same incidence of MUA (Wied et al. 2015).

**Table 1.** Reported incidences of MUA in the literature

Author	Setting	Country	TKA, n	MUA (%)
Kelly et al. 2018	3 centers from the	USA	5,520	0.5
	Kaiser Permanente TJRR			
Yoo et al. 2015	Single center	Korea	4,449	0.5
Pagoti et al. 2018	Single surgeon	UK	7,423	0.8
Pfefferle et al. 2014	Explory platform	USA	229,420	1.5
Namba et al. 2007	Kaiser Permanente TJRR	USA	9,640	2
Yeoh et al. 2012	Single center	UK	48	2.3
Bawa et al. 2013	Single center	USA	3,224	4.3
Werner et al. 2015	PearlDiver—database	USA	141,016	4.3
Ipach et al. 2011	Single center	Germany	858	4.5
Newman et al. 2018	Single center	USA	1,729	4.8
Issa et al. 2015	2 hospitals	USA	3,128	4.9
Wied et al. 2015	Fast-track hospital	Denmark	259	5.8
Pamilo et al. 2018	Fast-track hospital	Finland	624	5.9
Issa et al. 2014a	Single center	USA	2,128	6.8
Issa et al. 2014b	Single center	USA	1,973	7.3
Esler et al. 1999	Single center	UK	476	9.9

The risk of revision after MUA has been sparsely studied. Werner et al. (2015) found that patients who required MUA after TKA had an increased risk of revision while Pierce et al. (2017) did not find any increased risk in a matched case-control study including 138 patients.

We evaluated the incidence of 1st-time MUA performed within 1 year after primary total knee arthroplasty (TKA) and unicompartmental knee arthroplasty (UKA) surgery in Sweden, describe the demographics of the patients and the risk of revision.

## Patients and methods

We requested information on the 64,840 primary TKA (n = 61,835, 95.4%) and UKA (n = 3,005, 4.6%) that were registered in the Swedish Knee Arthroplasty Register (SKAR) between 2009 and 2013 from the Swedish National Board of Health and Welfare patient register (PAS). The SKAR has registered knee arthroplasties since 1975 and has a high completeness and correctness of data (SKAR 2018). The PAS contains in- and outpatient care, admission date, discharge date, surgical code (NOMESCO), diagnosis code (ICD-10), and operating hospital.

Based on the patient’s personal identification number (that includes information on date of birth and sex), contained in both registers, we requested information on knee arthroplasties registered in the PAS 2009–2014 with ICD-10 codes for joint stiffness (M24.5, M24.6, M25.6) together with the NOMESCO code for MUA (NGT19) or the codes for percutaneous, arthroscopic and open adhesiotomy (NGH30, NGH31, NGH32).

After receiving information from the PAS, the hospitals where the manipulations were performed were requested to provide medical records related to the manipulation in order to verify the side, diagnosis, NOMESCO code, surgical date, length of stay (LOS), and comorbidities. Further we had the intention to gather information from the records on the ROM before, during, and after the manipulation as well as the care after the manipulation.

Information on patient characteristics, age, sex, BMI, the ASA class, and history of prior knee surgery were obtained from the SKAR.

As the purpose of the study was to evaluate the incidence of MUA, we excluded percutaneous, arthroscopic, and open adhesiotomies, MUAs after revisions and reoperations, MUAs or primary knee arthroplasties performed outside the study period, duplicates, and those not verified as MUA. Further we included only the 1st-time MUAs performed within 1 year after the primary knee arthroplasty. The patients were followed-up until December 31, 2018. We use descriptive statistics and present the data in numbers and proportions.

### Statistics

Cumulative revision rate (CRR) curves were produced using the life table method with monthly intervals with 95% confidence intervals (CIs) calculated with the Wilson quadratic equation using the Greenwood and Peto effective sample size estimates (Dorey et al. 1993). When comparing the risk of age (continuous variable), sex, and MUA performed before or after 8 weeks from the primary knee arthroplasty, Cox regression was used to calculate relative risk estimates (RR) with CI. The reason for revision was presented as numbers and proportions.

### Ethics, funding, and potential conflicts of interests

The study was approved by the regional Ethics Committee of Stockholm (2015/978-31), and was performed in accordance with the Declaration of Helsinki. The study was not financed by any external funding. The authors declare no conflicts of interest.

## Results

**Figure 1.**
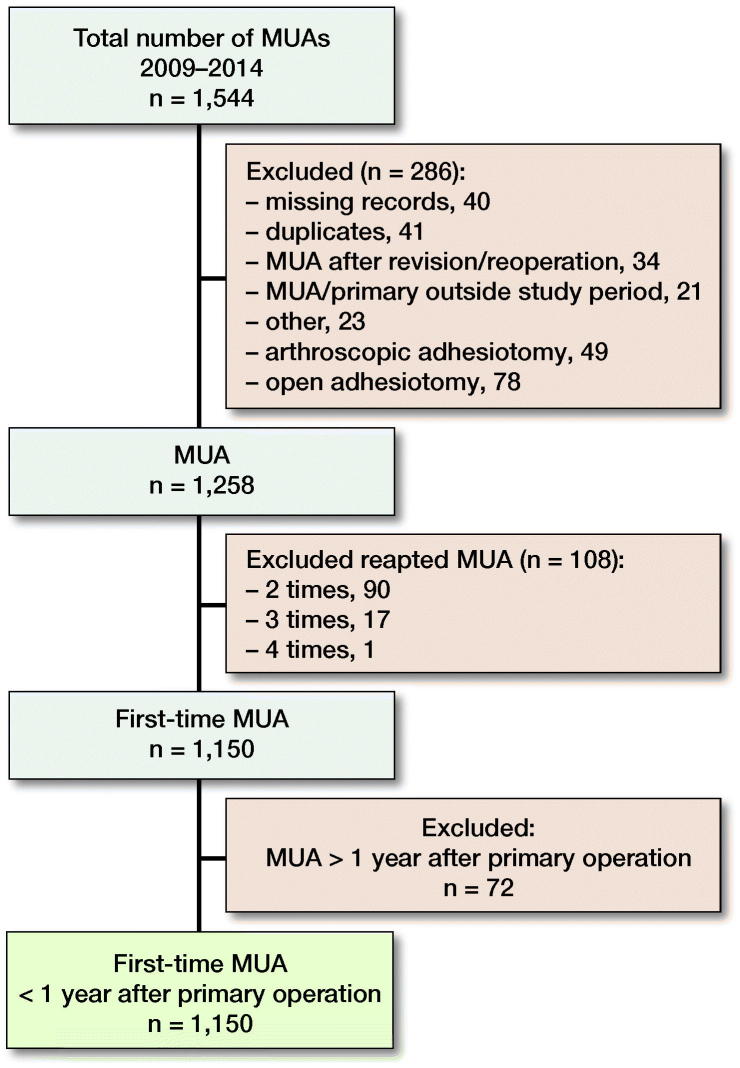
Flowchart of the study population.

We identified 1,258 MUAs of which 1,150 were 1st-time MUAs with 1,078 MUAs being performed within 1 year of the primary knee arthroplasty. All the hospitals (n = 75) responded to our request for medical records but in 40 cases (2.6%) a record could not be found (Figure 1).

**Figure 2.**
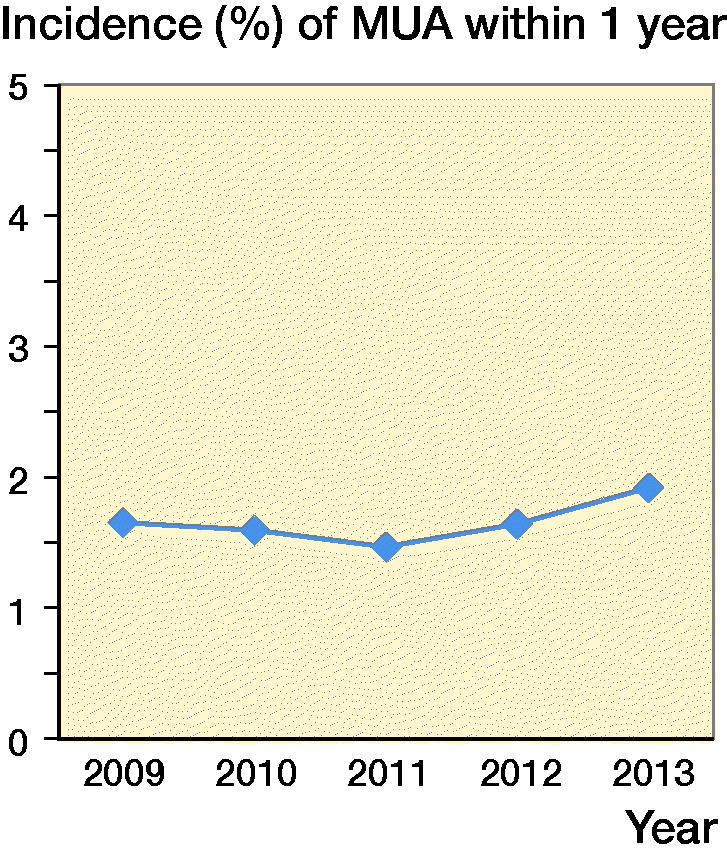
Incidence of MUA within 1 year in Sweden 2009–2013.

**Figure 3.**
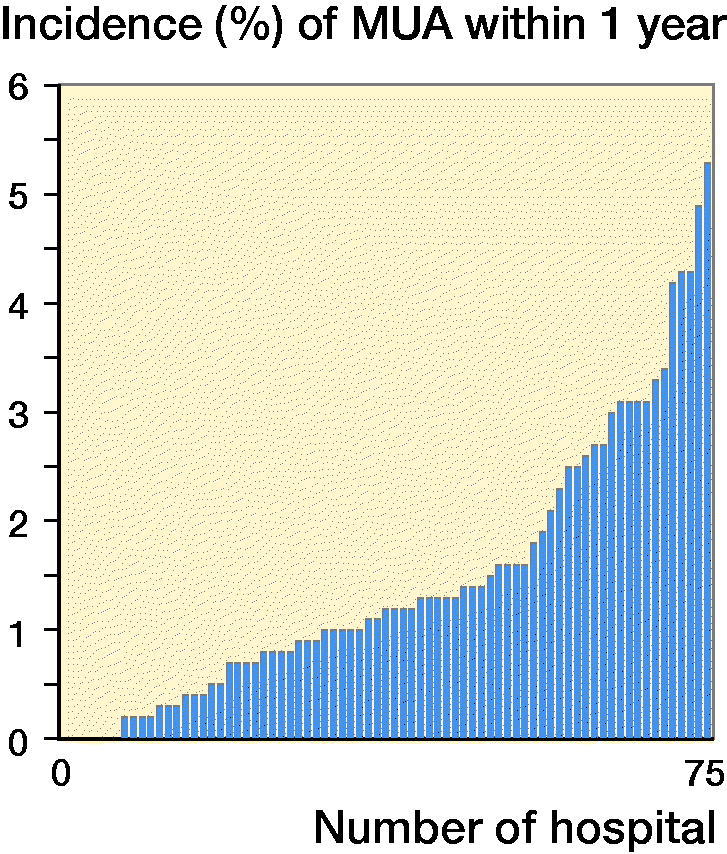
Incidence of MUA/hospital.

The incidence of 1st-time MUA within 1 year after knee arthroplasty was 1.7% and was similar through the years (Figure 2, see Supplemenatry data). The incidence of MUA varied between hospitals from 0% to 5% (Figure 3).

Of the TKAs, 1.7% (n = 1,061) underwent MUA and among the UKAs 0.6% (n = 17). Of the 1,078 MUAs, 60% were performed within 3 months after the primary knee arthroplasty.

The vast majority (n = 1,011, 94%) of the MUA patients were treated as inpatients and the median LOS was 2 days (0–20) with 95% of the patients staying 0–6 days.

As compared with the general knee arthroplasty population in Sweden, the MUA patients were younger (65% < 65 years), more often women, somewhat healthier and more often had a history of prior knee surgery. The BMI and diagnoses were similar (Table 2). 5.5% of the MUA patients were diagnosed with diabetes mellitus and 5.5% had warfarin treatment. Corresponding figures for the general arthroplasty population are not available.

**Table 2.** Characteristics of MUA patients and the general knee arthroplasty (TKA and UKA) population 2009–2013 in Sweden

	MUA	SKAR
Factor	(n = 1,154)	(n = 64,840)
Sex female, n (%)	729 (63)	37,490 (58)
Age, mean (SD)	61 (9)	69 (9)
ASA, n (%)^a^	1,136	63,440
I	336 (30)	12,345 (19)
II	671 (59)	41,010 (65)
III–IV	129 (11)	10,085 (16)
BMI^a^	1,133	63,347
mean (SD)	29 (5)	29 (5)
OA n (%)	1,091 (95)	62,042 (96)
Prior knee surgery^a^	1,125	62,934
n (%)	406 (36)	12,454 (20)

a Number of cases with data

Among the 1,078 MUAs there were 109 revisions. The CRR at 10 years was 10% (CI 8.6–12).

We found no statistically significant difference in risk of revision depending on sex (men hazard ratio [HR] 0.9 [CI 0.6–1.4]), age (HR 1 [CI 0.98–1]), or if the MUA was performed before or after 8 weeks following the knee arthroplasty surgery (< 8 weeks HR 1 [CI 0.6–1.6]). Femoro-patellar problems were the most common reason for revision (26%) followed by loosening and stiffness (Table 3, see Supplementary data).

**Table 3.** Numbers and proportions of the reasons for revision

Reason for revision	n
Femoro-patellar problems	28
Loosening	20
Stiffness	18
Infection	13
Suboptimal implant positioning	12
Instability	11
Progression of OA	5
Unspecified knee pain	1
Severe surgical error	1
Total	109

## Discussion

To our knowledge, this is the first nationwide study showing the incidence of MUA after primary knee arthroplasty surgery. All the hospitals answered our requests for medical records but 3% of the records could not be found and may have been misclassified. All privately run hospitals were represented in the PAS so we feel confident that the absolute majority of procedures were captured. However, unfortunately the medical records proved to be insufficient to evaluate the exact ROM before, during, and after MUA, regarding the change in ROM achieved during or after the MUA, or whether the patients were satisfied with the results.

We found the incidence of a 1st-time MUA within 1 year of knee arthroplasty surgery to be 1.7%, i.e., a rather uncommon procedure. Our incidence may be regarded to be low as compared with what has most commonly been reported (see Supplementary data). The 2 Nordic studies (Wied et al. 2015, Pamilo et al. 2018) included patients operated with TKA during the same time period as the patients in our study, but in fast-track hospitals. These studies showed an incidence of MUA of almost 6%, which was comparable to the hospital in Sweden with the highest incidence (5%). The SKAR has no information on whether the hospitals consider themselves as fast-track hospitals or not. On the other hand, we could not see a difference in incidence between government and private-run or high- and low-volume hospitals. However, the incidence varied between hospitals in Sweden in a similar way to the incidences from different hospitals in the literature (Table 1). This may reflect the highlighted lack of clear indications for MUA after knee arthroplasty surgery.

Several variables have been suggested as potential risk factors for stiffness requiring MUA, but little consensus exists (Kornuijt et al. 2018). The Swedish MUA population was more often women, younger, and somewhat healthier but had a higher proportion of previous knee surgery compared twith the general knee arthroplasty population. Diabetes and warfarin treatment have been suggested as potential risk factors (Issa et al. 2014b, Pfefferle et al. 2014) for joint stiffness but that information is not available in the SKAR and we do not know if they are over-represented in the Swedish MUA population.

The optimal timing of MUA is unknown. Early intervention has been suggested to be favorable (Bawa et al. 2013, Issa et al. 2014a, Desai et al. 2014, Ferrel et al. 2015, Vanlommel et al. 2017, Newman et al. 2018) while others found no difference between early and late intervention (Ipach et al. 2011, Yeoh et al. 2012). However, early intervention has been reported to vary from ≤ 6 to 20 weeks in the above-mentioned studies.

Werner et al. (2015) found that the risk of revision was less common in patients who underwent MUA within 8 weeks after the primary TKA (94/2,465 [3.8%]) as compared with patients who underwent MUA between 8 weeks and 3 months after (99/1,870 [5.3%]), p = 0.02. However, we found no statistically significant difference in risk of revision depending on whether the MUA was performed ≤ 8 or > 8 weeks. The reasons may be the difference in the number of MUAs in the studies and that our time limit for “late” MUAs was not 3 months but 1 year.

Further, we found that the MUA patients had approximately double the 10-year CRR of the general knee arthroplasty population in Sweden (SKAR 2018). This is in line with the findings of Werner et al. (2015), who found that patients who required MUA after the primary TKA had increased risk of revision (4.8%, within 7 years) as compared with those not requiring MUA (2%) (OR 2.4, CI 2.1–2.8).

Of the 109 revisions, 18 were due to stiffness and the rest for other reasons, and may not have had anything to do with the stiffness/MUA. We feel confident with the reasons for revision as we routinely read all the surgical records and the discharge letters at the register. We found that femoro-patellar problems comprised the most common reason for revision in our MUA cohort (26%) with infection (12%) being the 4th most common. Werner et al. (2015) reported only on the frequency of infections as reason for revision and found them to account for 16%.

The relatively large variation in the incidence between hospitals in Sweden may indicate that factors other than known risk factors such as sex, age, health, and postoperative ROM influence the decision to perform MUA. Rather, the decision may to a larger extent be affected by the patient’s expectations and motivation as well as the surgeon’s expectations and willingness to perform MUA and not least the available resources in the hospitals concerned.

In summary, in Sweden, MUA is a rather uncommon measure after knee arthroplasty, especially after UKA, and has double the CRR at 10 years as compared with the general knee arthroplasty population. The frequency of the procedure varies between hospitals but in general MUA is performed more frequently in healthier and younger patients.

## Supplementary Material

Supplemental Material

## Supplementary data

Tables 1 and 3 and Figure 2 are available as supplementary data in the online version of this article, http://dx.doi.org/10.1080/ 17453674.2019.1637177

The study was conceived by MH, OR, and AWD. HT, NL, MH, and AWD obtained and compiled the information from the hospitals. OR and AWD performed the analyses. HT and AWD wrote the initial draft. All the authors contributed to the interpretation of the data and to revision of the manuscript.

The authors would like to thank the hospitals for their contribution to the study.

*Acta* thanks Walter van der Weegen and other anonymous reviewers for help with peer review of this study.

## References

[CIT0001] Bawa H S, Wera G D, Kraay M J, Marcus R E, Goldberg V M Predictors of range of motion in patients undergoing manipulation after TKA. Clin Orthop Relat Res 2013; 471(1): 258–63. 22968534 10.1007/s11999-012-2591-1 PMC3528912

[CIT0002] Desai A S, Karmegam A, Dramis A, Board N, Raut V Manipulation for stiffness following total knee arthroplasty: when and how often to do it? Eur J Orthop Surg Traumatol. 2014; 24(7): 1291–5. 24327007 10.1007/s00590-013-1387-7

[CIT0003] Dorey F, Nasser S, Amstutz H The need for confidence intervals in the presentation of orthopaedic data. J Bone Joint Surg Am 1993; 75(12): 1844–52. 8258558 10.2106/00004623-199312000-00017

[CIT0004] Esler C N, Lock K, Harper W M, Gregg P J Manipulation of total knee replacements. Is the flexion gained retained? J Bone Joint Surg Br 1999; 81(1): 27–9. 10067996 10.1302/0301-620x.81b1.8848

[CIT0005] Ferrel J R, Davis R L 2nd, Agha OA, Politi J R Repeat manipulation under anesthesia for persistent stiffness after total knee arthroplasty achieves functional range of motion. Surg Technol Int 2015; 26: 256–60. 26055017

[CIT0006] Gadinsky N E, Ehrhardt J K, Urband C, Westrich G H Effect of body mass index on range of motion and manipulation after total knee arthroplasty. J Arthroplasty 2011; 26(8): 1194–7. 21277161 10.1016/j.arth.2010.12.004

[CIT0007] Ipach I, Mittag F, Lahrmann J, Kunze B, Kluba T Arthrofibrosis after TKA: influence factors on the absolute flexion and gain in flexion after manipulation under anaesthesia. BMC Musculoskelet Disord 2011; 12: 184. 21838865 10.1186/1471-2474-12-184 PMC3175211

[CIT0008] Issa K, Banerjee S, Kester M A, Khanuja H S, Delanois R E, Mont M A The effect of timing of manipulation under anesthesia to improve range of motion and functional outcomes following total knee arthroplasty. J Bone Joint Surg Am 2014a; 96(16): 1349–57. 25143495 10.2106/JBJS.M.00899

[CIT0009] Issa K, Kapadia B H, Kester M, Khanuja H S, Delanois R E, Mont M A Clinical, objective, and functional outcomes of manipulation under anesthesia to treat knee stiffness following total knee arthroplasty. J Arthroplasty 2014b; 29(3): 548–52. 24011781 10.1016/j.arth.2013.07.046

[CIT0010] Issa K, Rifai A, Boylan MR, Pourtaheri S, McInerney V K, Mont M A Do various factors affect the frequency of manipulation under anesthesia after primary total knee arthroplasty? Clin Orthop Relat Res 2015; 473(1): 143–7. 25002219 10.1007/s11999-014-3772-x PMC4390931

[CIT0011] Keating E M, Ritter M A, Harty L D, Haas G, Meding J B, Faris P M, Berend M E Manipulation after total knee arthroplasty. J Bone Joint Surg Am 2007; 89(2): 282–6. 17272441 10.2106/JBJS.E.00205

[CIT0012] Kelly M P, Prentice H A, Wang W, Fasig B H, Sheth D S, Paxton E W Reasons for ninety-day emergency visits and readmissions after elective total joint arthroplasty: results from a US integrated healthcare system. J Arthroplasty 2018; 33(7): 2075–81. 29523446 10.1016/j.arth.2018.02.010

[CIT0013] Kim J, Nelson C L, Lotke P A Stiffness after total knee arthroplasty: prevalence of the complication and outcomes of revision. J Bone Joint Surg Am 2004; 86-A(7): 1479–84. 15252096

[CIT0014] Kornuijt A, Das D, Sijbesma T, de Vries L, van der Weegen W Manipulation under anesthesia following total knee arthroplasty: a comprehensive review of literature. Musculoskelet Surg 2018; 102(3): 223–30. 29546693 10.1007/s12306-018-0537-9

[CIT0015] Namba R S, Inacio M Early and late manipulation improve flexion after total knee arthroplasty. J Arthroplasty 2007; 22(6 Suppl. 2): 58–61. 17823017 10.1016/j.arth.2007.02.010

[CIT0016] Newman E T, Herschmiller T A, Attarian D E, Vail T P, Bolognesi M P, Wellman S S Risk factors, outcomes, and timing of manipulation under anesthesia after total knee arthroplasty. J Arthroplasty 2018; 33(1): 245–9. 28935340 10.1016/j.arth.2017.08.002

[CIT0017] Pagoti R, O’Brien S, Blaney J, Doran E, Beverland D Knee manipulation for reduced flexion after total knee arthroplasty: is timing critical? J Clin Orthop Trauma 2018; 9(4): 295–9. 30449974 10.1016/j.jcot.2017.11.017 PMC6224686

[CIT0018] Pamilo K J, Torkki P, Peltola M, Pesola M, Remes V, Paloneva J Fast-tracking for total knee replacement reduces use of institutional care without compromising quality. Acta Orthop 2018; 89(2): 184–9. 29160123 10.1080/17453674.2017.1399643 PMC5901516

[CIT0019] Pfefferle K J, Shemory S T, Dilisio M F, Fening S D, Gradisar I M Risk factors for manipulation after total knee arthroplasty: a pooled electronic health record database study. J Arthroplasty 2014; 29(10): 2036–8. 24927868 10.1016/j.arth.2014.05.001

[CIT0020] Pierce T P, Issa K, Festa A, Scillia A J, McInerney V K, Mont M A Does manipulation under anesthesia increase the risk of revision total knee arthroplasty? A matched case control study. J Knee Surgery 2017; 30(7): 730–3. 10.1055/s-0037-1598040 28196393

[CIT0021] Plate J F, Wohler A D, Brown M L, Sun D, Fino N F, Lang J E Factors associated with range of motion recovery following manipulation under anesthesia. Surg Technol Int 2016; 29: 295–301. 27728948

[CIT0022] Ritter M A, Harty L D, Davis K E, Meding J B, Berend M E Predicting range of motion after total knee arthroplasty: clustering, log-linear regression, and regression tree analysis. J Bone Joint Surg Am 2003; 85-A(7): 1278–85. 10.2106/00004623-200307000-00014 12851353

[CIT0023] SKAR The Swedish Knee Arthroplasty Register Annual Report 2018. 2018; ISBN 978-91-88017-19-2.

[CIT0024] Vanlommel L, Luyckx T, Vercruysse G, Bellemans J, Vandenneucker H Predictors of outcome after manipulation under anaesthesia in patients with a stiff total knee arthroplasty. Knee Surg Sports Traumatol Arthrosc 2017; 25(11): 3637–43. 28032122 10.1007/s00167-016-4413-6

[CIT0025] Werner B C, Carr J B, Wiggins J C, Gwathmey F W, Browne J A Manipulation under anesthesia after total knee arthroplasty is associated with an increased incidence of subsequent revision surgery. J Arthroplasty 2015; 30(9 Suppl): 72–5. 26071252 10.1016/j.arth.2015.01.061

[CIT0026] Wied C, Thomsen M G, Kallemose T, Myhrmann L, Jensen L S, Husted H, Troelsen A The risk of manipulation under anesthesia due to unsatisfactory knee flexion after fast-track total knee arthroplasty. Knee 2015; 22(5): 419–23. 25766466 10.1016/j.knee.2015.02.008

[CIT0027] Yeoh D, Nicolaou N, Goddard R, Willmott H, Miles K, East D, Hinves B, Shepperd J, Butler-Manuel A Manipulation under anaesthesia post total knee replacement: long term follow up. Knee 2012; 19(4): 329–31. 21703859 10.1016/j.knee.2011.05.009

[CIT0028] Yoo J H, Oh J C, Oh H C, Park S H Manipulation under anesthesia for stiffness after total knee arthroplasty. Knee Surg Relat Res 2015; 27(4): 233–9. 26676186 10.5792/ksrr.2015.27.4.233 PMC4678244

