# Supplementary material for: Manipulation under anesthesia after primary knee arthroplasty in Sweden: incidence, patient characteristics and risk of revision
Source: Acta Orthop. 2019 Jul 4;90(5):484–8. doi: 10.1080/17453674.2019.1637177 (PMC6746267; doi:10.1080/17453674.2019.1637177)
Supplement: Supplemental Material [file IORT_A_1637177_SM7677.pdf]

Supplementary data

Table 1. Reported incidences of MUA in the literature

Author	Setting	Country	TKA, n	MUA (%)
Kelly et al. 2018	3 centers from the Kaiser Permanente TJRR	USA	5,520	0.5
Yoo et al. 2015	Single center	Korea	4,449	0.5
Pagoti et al. 2018	Single surgeon	UK	7,423	0.8
Pfefferle et al. 2014	Explory platform	USA	229,420	1.5
Namba et al. 2007	Kaiser Permanente TJRR	USA	9,640	2
Yeoh et al. 2012	Single center	UK	48	2.3
Bawa et al. 2013	Single center	USA	3,224	4.3
Werner et al. 2015	PearlDiver—database	USA	141,016	4.3
Ipach et al. 2011	Single center	Germany	858	4.5
Newman et al. 2018	Single center	USA	1,729	4.8
Issa et al. 2015	2 hospitals	USA	3,128	4.9
Wied et al. 2015	Fast-track hospital	Denmark	259	5.8
Pamilo et al. 2018	Fast-track hospital	Finland	624	5.9
Issa et al. 2014a	Single center	USA	2,128	6.8
Issa et al. 2014b	Single center	USA	1,973	7.3
Esler et al. 1999	Single center	UK	476	9.9

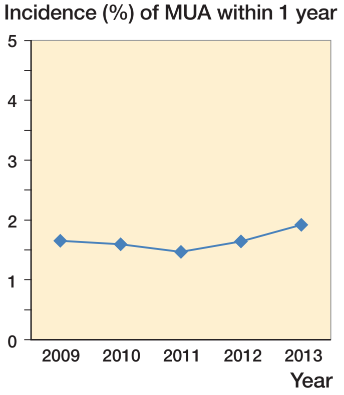

Figure 2. Incidence of MUA within 1 year in Sweden 2009–2013.

Table 3. Numbers and proportions of the reasons for revision

Reason for revision	n
Femoro-patellar problems	28
Loosening	20
Stiffness	18
Infection	13
Suboptimal implant positioning	12
Instability	11
Progression of OA	5
Unspecified knee pain	1
Severe surgical error	1
Total	109
